# Prognostic value of quantitative flow ratio in patients with coronary heart disease after percutaneous coronary intervention therapy: a meta-analysis

**DOI:** 10.3389/fcvm.2023.1164290

**Published:** 2023-08-07

**Authors:** Huaigang Chen, Lang Hong, Gang Xi, Hong Wang, Jing Hu, Qi Liu, Liu Yang

**Affiliations:** ^1^ Medical College of Nanchang University, Nanchang, China; ^2^ Department of Cardiology, Jiangxi Provincial People’s Hospital, The First Affiliated Hospital of Nanchang Medical College, Nanchang, China; ^3^ Department of Cardiology, The Third People's Hospital of Jingdezhen, Jingdezhen, China

**Keywords:** coronary heart disease, percutaneous coronary intervention, prognosis, meta, quantitative flow ratio

## Abstract

**Background:**

Coronary atherosclerotic heart disease is one of the most serious health and life-threatening diseases. There is no doubt that despite the increasing number of assessment methods used clinically, the prognosis assessment is still not ideal, and newer assessment methods are needed.

**Objective:**

To investigate the predictive value of quantitative flow ratio (QFR) for adverse events (vessel-oriented composite endpoint events/target lesion failure) in patients after percutaneous coronary intervention (PCI).

**Method:**

Eight studies involving 4,173 patients (5,688 vascular lesions) were included. These are studies on the relationship between QFR values and prognosis of adverse cardiac events after PCI. This meta-analysis was performed after quality assessment and data extraction of clinical trials data that met the inclusion criteria.

**Result:**

Each of the eight studies described the cut-off values for the best predictive ability of post-PCI QFR and the hazard ratio (HR) between QFR values and adverse events, respectively. The pooled HR of these studies was 4.72 (95% CI: 3.29–6.75). Concurrently, lower post-PCI QFR values were associated with the occurrence of individual clinical events (cardiac death/myocardial infarction/target vessel revascularization), with relative risk values of 6.51 (95% CI: 4.96–8.53), 4.83 (95% CI: 3.08–7.57), and 4.21 (95% CI: 2.66–6.68), respectively.

**Conclusion:**

QFR may have great potential in the assessment of prognosis. It is necessary to measure QFR value after PCI. A lower QFR value after PCI was an important predictor for experiencing adverse events.

## 1. Introduction

Coronary atherosclerotic heart disease (CAD) is still one of the most serious diseases endangering human health and life (1). The most effective treatment of coronary heart disease is percutaneous coronary intervention (PCI), which can significantly restore blood perfusion against myocardial ischemia. As we all know, common methods of PCI include stent implantation and drug-coated balloon (DCB) (2). Obviously, the ultimate goal of any treatment method is to increase PCI success rate and thus improve the prognosis of patients. At present, there have been many methods for determining the prognosis of patients receiving PCI. The most commonly used method is thrombolysis in myocardial infarction (TIMI) blood flow grading; however, this assessment thus has limitations in both precision and objectivity especially depending on the experience of the interventional cardiologists. Hence, the TIMI blood flow score can vary widely, even in the same patient (3). Moreover, the European system for cardiac operative risk evaluation 2 (EuroSCORE 2) can be used to predict in-hospital mortality by evaluating patients after PCI through 18 clinical characteristics, which may overestimate it (4, 5). Also, the use of SYNTAX Ⅱ score is limited to the long-term mortality of revascularization for patients with complex three-vessel coronary artery disease and left main coronary artery involvement (6, 7). Unquestionably, although more and more assessment methodologies are used in clinics, the evaluation of prognosis is still not ideal.

More updated evaluation methods are needed to improve the outcomes. At present, new and effective methods are appearing, for instance, quantitative flow ratio (QFR), fractional flow reserve (FFR), etc. Among them, FFR is a physiological evaluation index. This is the gold standard for assessing the physiological severity of coronary stenosis (8). According to two recent meta-analyses, impaired fractional flow reserve following percutaneous coronary intervention is a prevalent condition after drug-eluting stent deployment (9, 10). This condition independently predicts the occurrence of target vessel revascularization (TVR) as well as cardiac mortality or myocardial infarction (MI). However, this method is expensive, time-consuming, and risky. At the same time, QFR is emerging as an effective prognostic evaluation method. Quantitative flow ratio, which is based on coronary angiography (CA) images, is a method for rapidly calculating FFR from blood flow velocity contrasts during three-dimensional quantitative coronary angiography (3D-QCA) (11). By using CA as a reference standard, the diagnostic role of QFR in assessing the degree of coronary stenosis has been demonstrated in many studies (12–14). In recent years, some studies on the prognostic value of QFR in patients after PCI have been presented (15–22); however, no one has systematically studied this problem. The aim of this meta-analysis was to evaluate the prognostic value of QFR in patients with coronary heart disease after PCI therapy.

## 2. Materials and methods

### 2.1. Search strategy

Two researchers, HC and LY, searched PubMed, EMBASE, clinical controlled trial database of the Cochrane Library, and Sino-med databases. The search period was from the establishment of the database to March 2022. The keywords are as follows: QFR, quantitative flow ratio, QFR and CAD, QFR and Coronary atherosclerotic disease, quantitative flow ratio and Coronary atherosclerotic disease, quantitative flow ratio and coronary heart disease. Simultaneously, we have outlined detailed search strategies (Supplementary Material S1).

### 2.2. Inclusion and exclusion criteria for the literature study

The inclusion criteria were as follows: (1) types of study: randomized controlled study, cohort study, case–control study; (2) subjects: patients with coronary heart disease who underwent interventional therapy (stent implantation or drug-coated balloon); (3) observation indicators: the QFR value was measured and recorded after interventional therapy; (4) outcomes: outcomes or prognosis during follow-up were observed, mainly vessel-oriented composite endpoint events (VOCE), which were defined as composite of cardiac death, MI, TVR, major adverse cardiac events (MACE), and target lesion failure (TLF); (5) the period of publication literature was from the establishment of the database to March 2022, and there are no restrictions for this research.

The exclusion criteria were as follows: (1) repeated published literature; (2) study presented no outcome indicators; (3) only abstract or conference abstract (incomplete information provided); (4) study population consisting of CAD patients without any history of PCI; (5) literature reviews, reviews, expert comments, animal experiments or basic experiments, etc. (6) studies with less than 20 included patients or rate of loss to follow-up was more than 20%.

### 2.3. Literature screening

Two researchers, HC and LY, independently extracted data from each study. If no agreement can be reached, another author would mediate.

### 2.4. Quality evaluation and data extraction

The modified Jadad scale was used to evaluate the quality of randomized controlled trials in this meta-analysis (23). The evaluation indicators included random sequence generation, randomization, allocation concealment, and blinding. It is a 7-point system with values ≥4 as high quality and ≤3 as low quality. The quality of the cohort study was evaluated using the Newcastle–Ottawa Scale (NOS) (24). The NOS scale assesses the quality of research by assessing three modules consisting of eight items. These modules include the selection of the study population, comparability, and the assessment of the outcome. It should be noted that the total score of this scale is 9, meaning that the higher the score, the better the quality of the literature.

Moreover, the variables extracted from the original studies were summarized as follows: study design, demographics, clinical presentation, follow-up duration, QFR measurements, and clinical events (including primary endpoint events, secondary outcomes, and their definitions) with hazard ratios (HRs) and 95% confidence intervals (CIs). All data were manually extracted by two researchers and organized into tables.

### 2.5. Data analysis

Meta-analysis was conducted by Stata 17.0 software. As for categorical variables, we select odds ratio (OR), relative risk (RR), or HR. The logHR and SElogHR and a pooled HR value were calculated according to the formula. The enumeration data were evaluated by standardized mean difference (SMD) and weighted mean difference (WMD). Both were presented with 95% CIs. The chi-square test was used to examine heterogeneity among the results of the included studies. If there is no statistical heterogeneity (*P* > 0.10, *I*^2^ < 50%) and clinical heterogeneity in the research results, we will use a fixed-effect model for meta-analysis. Otherwise, the causes of heterogeneity are analyzed first, and the random effect model is used for meta-analysis. At the same time, the possible causes of heterogeneity are found out from both clinical and methodological aspects. For trials that were clinically heterogeneous or presented insufficient information for pooling, we provide a descriptive analysis. We chose to present the results of the analysis graphically using a forest plot.

## 3. Results

### 3.1. Literature search results

**Figure 1:**
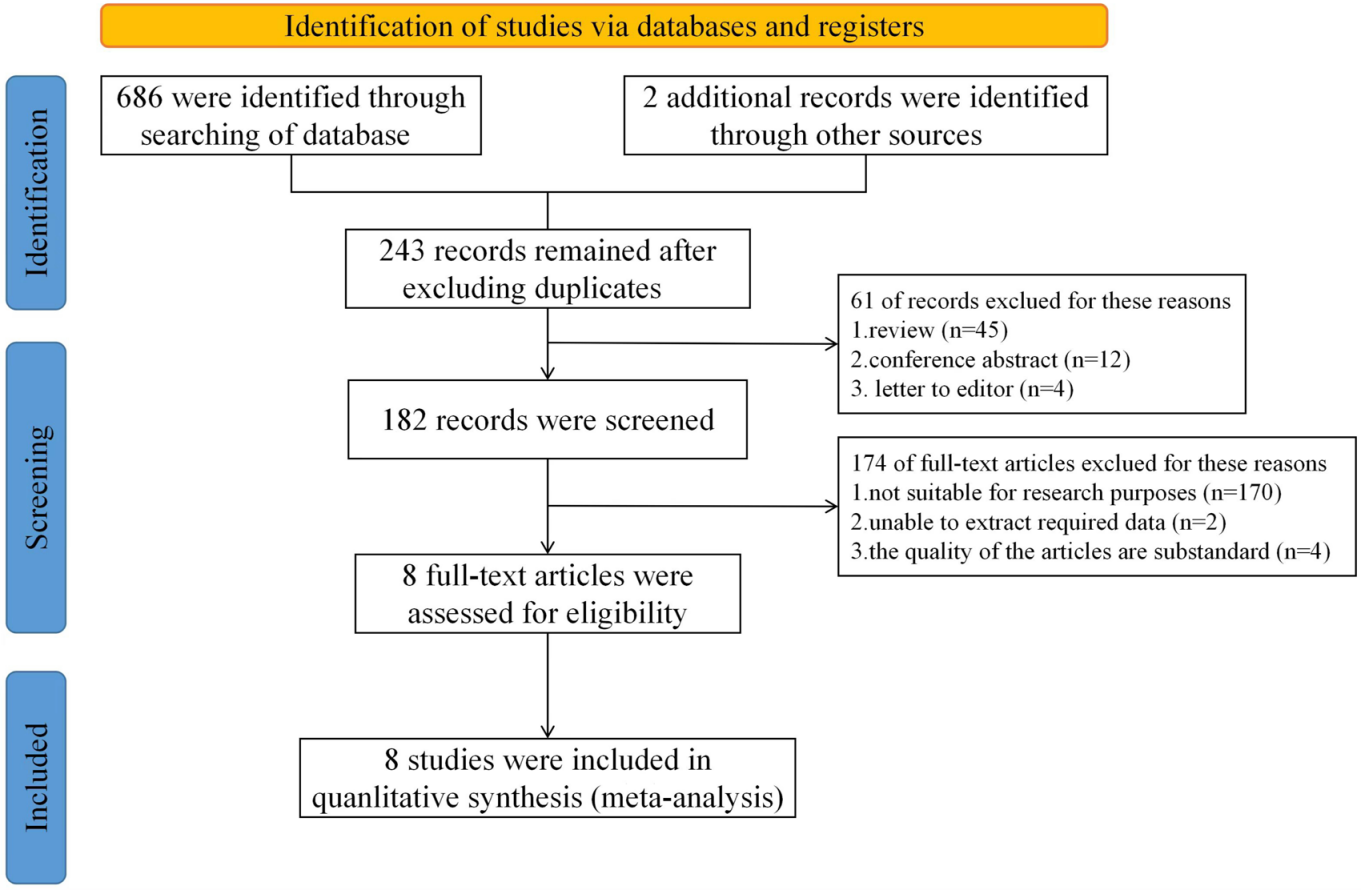
Flow diagram of search and study selection.

According to the proposed search terms, each database was searched respectively. A total of 243 related literature studies were initially detected, wherein the publication year was from 2001 to 2022. After reading the title and abstract, 61 literature studies were excluded as they were unrelated to the research question. After further full-text reading, another 172 articles were excluded. Finally, eight literature studies were included in this study. The flow chart is shown in Figure 1. A total of 4,173 patients were included in eight studies, out of which 1,109 were in the low QFR group and the rest of were in the high QFR group (*n* = 4,579). According to the modified Jadad scale, both studies were RCTs and had a high-quality rating, with scores greater than 3. On the other hand, according to the NOS scale, the results showed that seven studies received a high rating (scores ≥8) (Supplementary Material S2).

### 3.2. Publication bias

**Figure 2:**
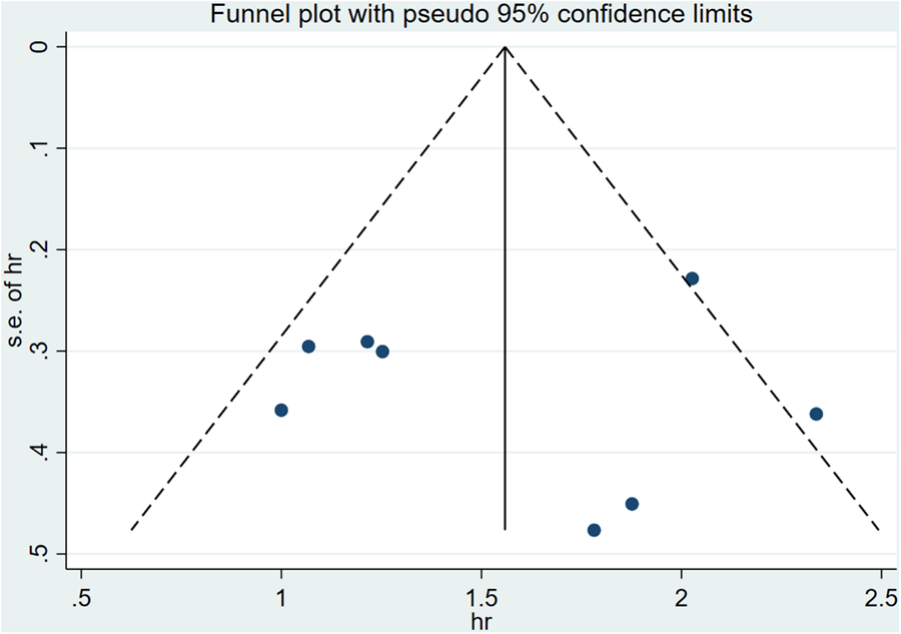
Funnel plot of publication bias among the included studies.

A funnel plot was performed to assess the publication bias of literatures (Figure 2), showing that the literature studies included are less likely to have a publication bias. The risk of adverse prognostic events was higher in the low QFR value group than those in the high QFR value group.

### 3.3. Data analysis results

#### 3.3.1. Main clinical features

The main clinical features of patients in each trial are shown in Table 1. Overall, the mean age of patients was 63.6 years, 74.6% were male, 25.9% had diabetes mellitus (DM), and 45% had left anterior descending (LAD) artery disease. Characteristics at baseline and lesion location were well balanced between groups [sex, diabetes, hyperlipidemia, hypertension, and body mass index (BMI)] (*P* > 0.05). Table 2 shows that the mean follow-up time for each study was 2.22 years, the mean size of the cut-off value was 0.90, and the primary endpoint event was made the one-to-one correspondence for each trial. Furthermore, the QFR analysis in all included studies was performed in a core laboratory in an offline mode. As shown in Table 3, the specific number of occurrences of the three secondary clinical events of interest in this study, the optimal cut-off values ranged from 0.89 to 0.94. Among these, in the lower QFR group, the incidence of target vessel revascularization events was markedly higher compared to the high QFR group (15% vs. 2%).

**Table 1:** Baseline patient characteristics and lesion location.

Study	Patients	Vessels	Age (years)	Male (%)	Population (%)	BMI (kg/m^2^)	Hyperlipidemia (%)	Hypertension (%)	Diabetes (%)	Distribution (%)		
										LAD	LCX	RCA
Kogame et al. (16)	602	751	68	443 (74)	European	26.5	336 (56)	444 (74)	139 (23)	356 (48)	184 (24)	211 (28)
Biscaglia et al. (15)	393	771	66.6	364 (92.7)	European	29.0	297 (77.1)	295 (79.4)	116 (29.7)	352 (45.7)	243 (31.5)	176 (22.8)
Tang et al. (17)	186	415	63.1	140 (75.3)	Asian	24.9	35 (18.8)	115 (61.8)	65 (34.9)	169 (40.7)	106 (25.5)	140 (33.7)
Bar et al. (19)	617	946	61.9	474 (76.8)	European	27.1	348 (56.4)	284 (46)	86 (13.9)	254 (26.9)	464 (49)	228 (24.1)
Tang et al. (18)	177	185	68	143 (80.8)	Asian	25.9	35 (19.8)	131 (74)	84 (47.5)	93 (50.3)	37 (20)	55 (29.7)
Liu et al. (20)	169	169	62.5	128 (75.7)	Asian	25.5	58 (34.3)	124 (73.4)	69 (40.8)	81 (47.9)	25 (14.8)	63 (37.3)
You et al. (21)	224	224	71.1	152 (67.9)	Asian	NR	155 (69.2)	168 (75)	91 (40.6)	177 (79)	12 (5.4)	35 (15.6)
Zhang et al. (22)	1,805	2,227	60.9	1,268 (70.2)	Asian	24.9	577 (32)	1,119 (62)	431 (23.9)	1,078 (48.4)	481 (21.6)	668 (30)
Overall	4,173	5,688	63.6	3,112 (74.6)		26.0	1,841 (44.1)	2,680 (64.2)	1,081 (25.9)	2,560 (45)	1,552 (27.3)	1,576 (27.7)

LCX, left circumflex; NR, not reported; RCA, right coronary artery.

Values are mean or number (%).

**Table 2:** The results summary of clinical outcomes and analyses.

Study	Follow-up time (years)	Cut-off value	Sensitivity	Specificity	AUC	Model	Outcome event	Number of events	HR	HR (95% CI)
								≤Cut-off value	>Cut-off value	
Kogame et al. (16)	1.75	0.89	60%	87%	0.77	Offline	VOCE	31/123 (25%)	22/628 (3.5%)	2.91 (1.63–5.19)
Biscaglia et al. (15)	2	0.91	65%	64%	0.702	Offline	VOCE	34/284 (12%)	18/487 (3.7%)	3.37 (1.91–5.97)
Tang et al. (17)	2	0.91	NR	NR	0.72	Offline	VOCE	21/101 (20.8%)	18/314 (5.7%)	2.718 (1.347–5.486)
Bar et al. (19)	5	0.80	23.4%	97.5%	0.64	Offline	VOCE	13/36 (36.1%)	74/910 (8.1%)	3.50 (1.94–6.30)
Tang et al. (18)	1	0.94	74%	75%	0.77	Offline	VOCE	20/59 (33.9%)	7/126 (5.6%)	6.53 (2.7–15.80)
Liu et al. (20)	1	0.89	NR	NR	0.74	Offline	VOCE	11/36 (30.6%)	9/133 (6.8%)	5.94 (2.33–15.09)
You et al. (21)	3	0.94	89.5%	69.2%	0.826	Offline	TLF	36/54 (66.7%)	16/170 (9.4%)	10.35 (5.09–21.04)
Zhang et al. (22)	2	0.92	62.2%	83%	0.75	Offline	VOCE	51/416 (12.4%)	31/1,811 (1.7%)	7.59 (4.86–11.9)

NR, not reported.

**Table 3:** The results summary of clinical event occurrences.

Study	Cut-off value	Cardiac death		Myocardial infarction		Target vessel revascularization	
		≤Cut-off value	>Cut-off value	≤Cut-off value	>Cut-off value	≤Cut-off value	>Cut-off value
Kogame et al. (16)	0.89	4/284	4/487	4/284	4/487	30/284	14/487
Biscaglia et al. (15)	0.91	18/123	18/628	18/123	18/628	24/123	16/628
Tang et al. (18)	0.94	1/59	0/126	2/59	1/126	19/59	6/126
Liu et al. (20)	0.89	1/36	0/133	2/36	0/133	10/36	9/133
You et al. (21)	0.94	NR	NR	NR	NR	30/54	10/170
Zhang et al. (22)	0.92	15/416	11/1,811	9/416	10/1,811	33/416	15/1,811
Overall		39/918 (4.2%)	33/3,185 (1%)	35/918 (3.8%)	33/3,185 (1%)	146/972 (15%)	70/3,355 (2%)

NR, not reported.

#### 3.3.2. Predictive value of post-procedural QFR values for adverse events in CAD patients

**Figure 3:**
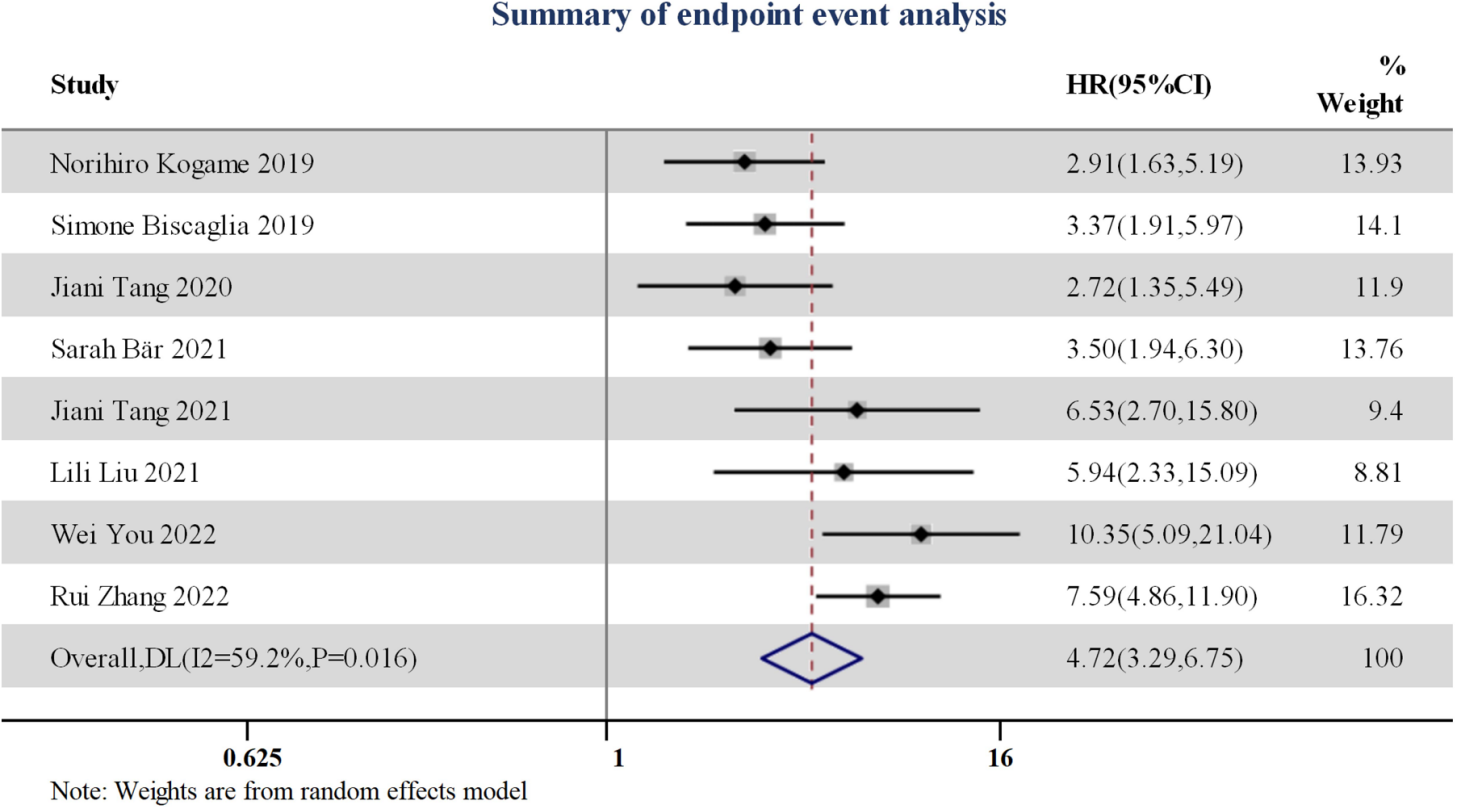
Forest plot for endpoint event summarization.

All studies included reported that post-procedural (or during follow-up period) QFR values, along with the best predictive ability cut-off value of QFR, with its respective sensitivity, specificity, and area under the curve (AUC). The HR and 95% confidence interval of adverse events (VOCE/TLF) was calculated from eight studies. The total combined HR value was 4.72 (95% CI: 3.29–6.75) (Figure 3). Patients in the high QFR group experienced more than four times the risk of adverse endpoint following the intervention, in contrast to those in the low QFR group.

**Figure 4:**
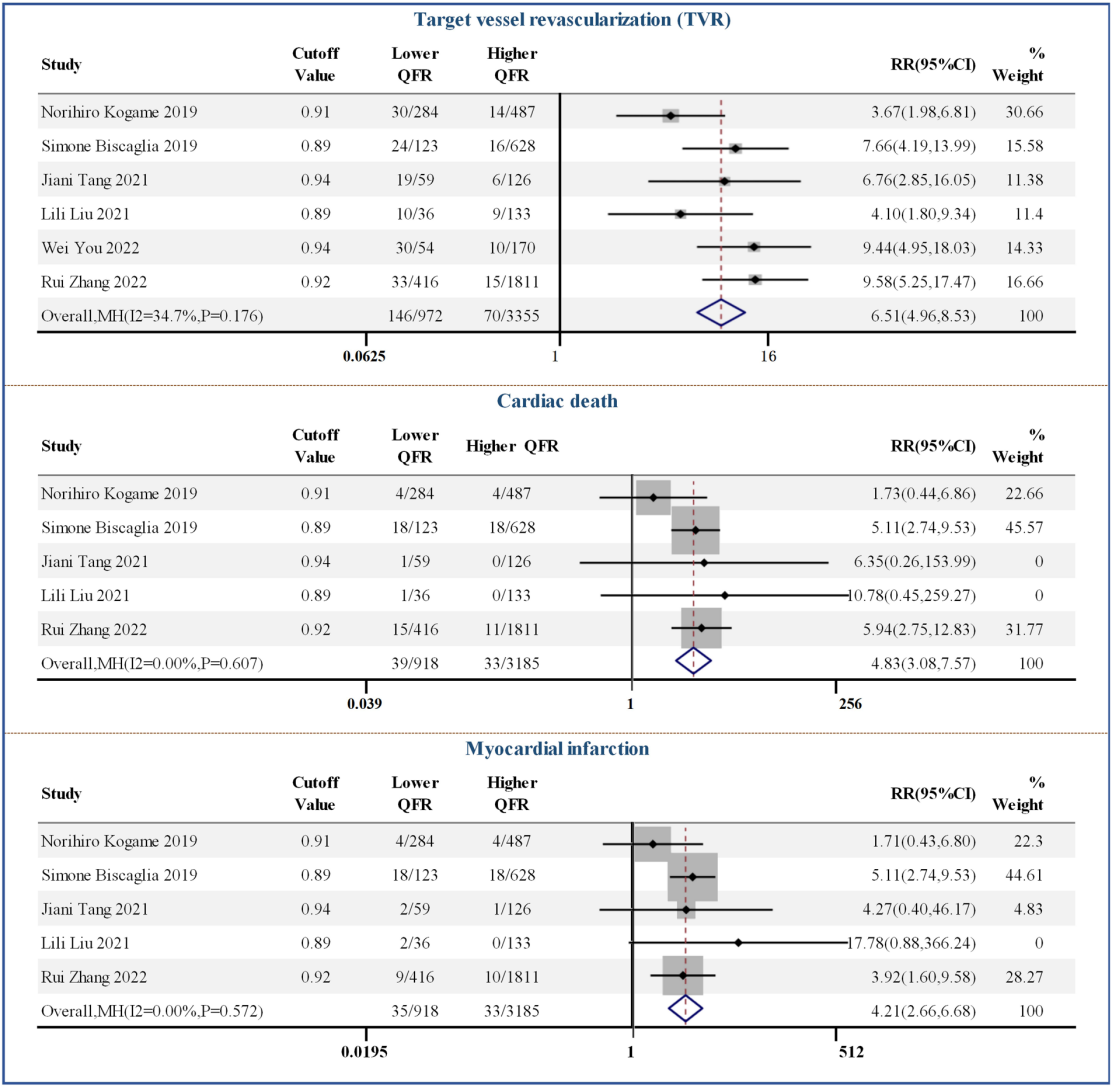
Forest plots of individual clinical events.

Figure 4 shows the results of the comparison between the post-PCI QFR threshold and the clinical event count. Six studies reported the specific number of TVR events after PCI, and the pooled RR was 6.51 (95% CI: 4.96–8.53). In addition, high QFR after PCI was beneficial for reducing the risk of cardiac death and MI when applying optimal cut-off values ranging from 0.89 to 0.92, with RR values of 4.83 (95% CI: 3.08–7.57) and 4.21 (95% CI: 2.66–6.68), respectively.

#### 3.3.3. Subgroup analyses

**Figure 5:**
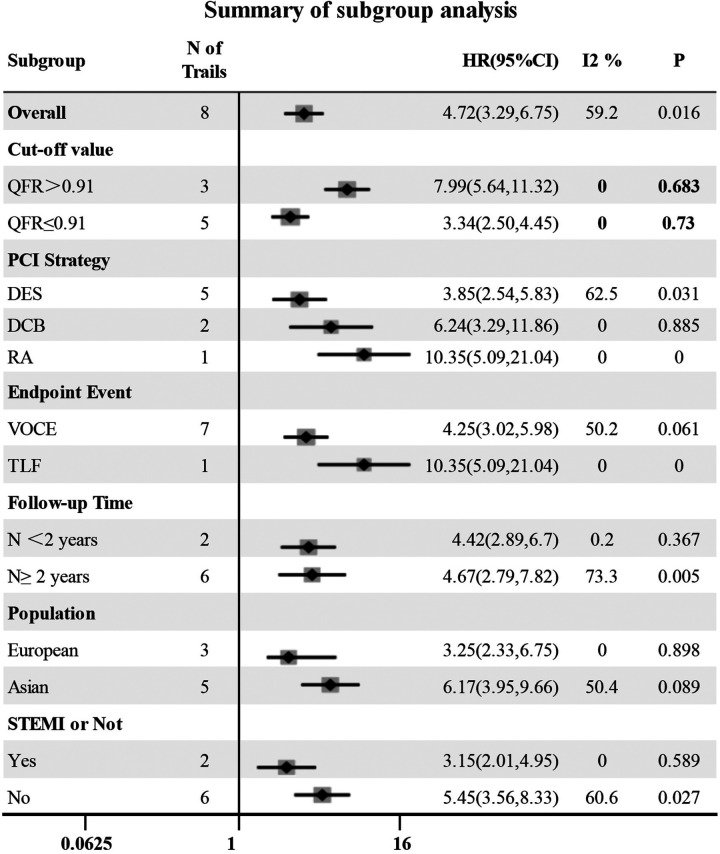
Forest plot for subgroup analysis. DES, drug stent implantation; RA, rotational atherectomy.

The QFR cut-off value, follow-up time, endpoint event, population, intervention strategy and whether it was ST-segment elevation myocardial infarction were considered possible sources of heterogeneity, and forest plots (Figure 5) were used to present all results. In the subgroup analysis, five and three studies were included in the high QFR group (QFR > 0.91) and the low QFR group (QFR ≤ 0.91), respectively, when QFR = 0.91 was used as the cut-off value. It was shown that the HR values of the low QFR group and the high QFR group were 3.34 (95% CI: 2.50–4.45) and 7.99 (95% CI: 5.64–11.32), respectively, and no significant heterogeneity was observed within the groups (*I*^2 ^< 50%, *P* > 0.1). However, significant heterogeneity was observed within the groups in other subgroups (*I*^2 ^> 50% or *P* < 0.1), indicating that follow-up time, endpoint event, population, intervention strategy, and whether it was STEMI were not the causes of heterogeneity in this study. A sensitivity analysis was also performed to verify the robustness of our study results, as shown in Figure 2.

In addition, QFR ≥ 0.89 was chosen as the cut-off value in seven (87.5%) of all the studies included, and the sensitivity and specificity were around 75%. Based on these data, we concluded that this may be a more credible threshold.

## 4. Discussion

### 4.1. Post-PCI QFR predicts the occurrence of adverse events

This was the first meta-analysis on the predictive value of post-PCI QFR, which pooled eight cohort studies and randomized controlled trials, including 4,173 patients and 5,688 vessel lesions, with follow-up time ranging from 1 to 5 years. The main findings of this study were as follows: (1) patients with low QFR value after PCI had a higher risk of VOCE and TLF during follow-up, with an HR value of 4.72 (95% CI: 3.29–6.75); (2) using various QFR cut-off values from 0.80 to 0.94, RR values showed that the lower QFR group after PCI had a higher risk of cardiac death, MI, and TVR.

On the other hand, QFR guidance has been proven to be superior to standard coronary angiography guidance (25). However, whether QFR measurement after PCI can effectively evaluate the treatment outcome and predict clinical adverse outcomes remains controversial. Even if angiography shows successful PCI, about 20% of vessels still have suboptimal post-PCI physiological status (26, 27). In recent years, more and more clinical studies have supported the prognostic value of post-PCI QFR. Biscaglia et al. and Kogame et al. conducted a more detailed stratification of the post-PCI QFR value of patients, so that we can more intuitively feel the difference in prognosis (15, 16). Similarly, their studies mentioned that patients with previous myocardial infarction, left anterior descending artery disease, and residual stenosis were the reasons for the low post-PCI QFR value. Kogame et al. also focused on more complex lesions (such as three-vessel disease) in coronary heart disease. Furthermore, all the post-PCI QFR values (vessels treated in the SYNTAXII trial) were retrospectively analyzed. Retrospective analysis performed by Tang et al. and Liu et al. included patients with in-stent restenosis after drug-coated balloon angioplasty (17, 20). In the meantime, the latest generation of QFR based on Murray's law (*μ*QFR) was applied. If we now turn to Tang et al. and Bar et al., they both shed light on STEMI patients (18, 19). Bar et al. defined a cut-off value of QFR = 0.80 in the study, which led to a low sensitivity of QFR (23.4%). You et al. investigated the clinical predictive value of QFRi (quantitative flow ratio in a segment) for the long-term outcome in patients who had heavily calcified lesions (21). These patients underwent PCI with rotational atherectomy. The result showed that lower QFRi post-PCI was associated with higher TLF, and the HR value is 10.35. It is the first to directly compare the clinical value of post-PCI QFR assessments in patients with and without DM by Zhang et al. (22). Their result showed that a higher post-PCI QFR value was associated with improved long-term prognosis regardless of the presence of DM, and HR value is higher in the DM cohort than in the non-DM cohort (6.24, 95% CI: 2.40–16.2, vs. 5.92, 95% CI: 3.28–10.7).

This prognosis result can be attributed to many pathophysiological reasons. Usually, common reasons for poorer prognosis are as follows. First, the presence of untreated stenosis, including diffuse non-significant stenosis outside the stent segment, which is the most common cause of residual pressure gradient after PCI (28, 29). Second, the position of the stent is not ideal (30–33). Finally, marginal dissection is also a factor affecting coronary blood flow. The results of Chung et al. showed that the FFR value was significantly lower with severe marginal dissection compared with mild marginal dissection (34). These functional data were used to define relevant outcomes. Quantitative flow ratio is precisely a novel, non-invasive, new method based on the functional data. Specifically, for untreated coronary artery stenosis and marginal dissection, QFR can accurately identify the index of the functional data. As for stent placement and unreasonable deployment, obviously, fluid hemodynamics is another determining factor in the pathophysiology of vascular lesions. *In vitro* studies have shown that local hemodynamic changes are one of the main factors determining the biological response of the vessel wall after stent implantation (35). In contrast, it is almost impossible to complete the evaluation of hemodynamics parameters by an operator. This may explain why QFR has such a meaningful value in predicting the prognosis of patients.

### 4.2. The optimal cut-off value of post-PCI QFR prediction

In addition, we found that the cut-off value of QFR was the main factor causing heterogeneity in this study. When QFR > 0.91 was used as the cut-off value, the risk of adverse events in the low QFR group was nearly eight times that of the high QFR group (HR = 7.99). However, when QFR ≤ 0.91 was used as the cut-off value, the risk ratio of outcome events between the two groups was 3.44. This can be explained as follows: the higher the post-PCI QFR value, the smaller the residual pressure gradient of the lesion (ideally, the post-PCI QFR value should be 1), reflecting good recovery of blood flow perfusion. The latest published data from the FORZA clinical trial showed that post-PCI QFR ≤ 0.89 was the only predictor of 3-year target vessel failure (TVF) occurrence after considering various factors such as smoking, age, and gender (36).

In conclusion, in view of the fact that prognosis models are limited in clinical practice, this meta-analysis suggests that among patients, QFR may have great potential in the assessment of prognosis. As a new non-invasive physiological evaluation index, QFR has many advantages such as fast calculation time and no use of drugs (adenosine). In addition to this, recent studies have reported that QFR also has good diagnostic efficacy in patients with coronary artery disease combined with severe aortic stenosis (AS), superior to the resting distal to aortic coronary pressure (Pd/Pa) ratio and instantaneous wave-free pressure ratio (iFR), when using FFR as a reference (37, 38). This technology has been continuously upgraded and improved, with AI techniques being added to the latest QFR (39). The indications of the QFR will be further expanded in the near future.

## 5. Limitation

Our meta-analysis has some limitations. The overall quality of the included studies was heterogeneous but relatively low, and most (six out of eight studies) were observational cohort studies. Therefore, the results of our meta-analysis should be interpreted with caution. Our study could only determine the correlation between post-PCI QFR value and prognosis by qualitative analysis. Various reasons prevented us from obtaining individual-level data from the original studies, which resulted in the inability to accurately calculate the optimal cut-off value of QFR. Second, the sample size of the current included studies was small, and future studies need to include large-scale, multicenter, prospective clinical trials.

## 6. Conclusion

QFR has potential in post-PCI prognosis evaluation, and lower post-PCI QFR value is an important predictor of adverse events and is associated with the future risk of cardiac death, MI, and TVR.

## Data Availability

The original contributions presented in the study are included in the article/Supplementary Material, further inquiries can be directed to the corresponding author.
