# Supplementary material for: Prognostic value of quantitative flow ratio in patients with coronary heart disease after percutaneous coronary intervention therapy: a meta-analysis
Source: Front Cardiovasc Med. 2023 Aug 7;10:1164290. doi: 10.3389/fcvm.2023.1164290 (PMC10441770; doi:10.3389/fcvm.2023.1164290)
Supplement: Supplementary file 1 [file Table1.docx]

**Supplement 2:**

Newcastle-Ottawa Scale for assessing the quality of studies in meta-analysis

|  | Kogame 2019 | Biscaglia 2019 | Jiani Tang 2020 | Sarah Bär 2021 | Jiani Tang 2021 | Lili Liu 2021 | Wei You 2022 | Rui Zhang 2022 |
| --- | --- | --- | --- | --- | --- | --- | --- | --- |
| **Selection** |  | | | | | | | |
| Representation of the exposure queue. | ★ | ★ | ★ | ★ | / | ★ | ★ | ★ |
| Selection of non-exposed queues | ★ | ★ | ★ | ★ | ★ | ★ | ★ | ★ |
| Determination of exposure | ★ | ★ | ★ | ★ | ★ | ★ | ★ | ★ |
| No study subjects already had the disease under study at the start of the study. | ★ | ★ | ★ | ★ | ★ | ★ | ★ | ★ |
| **Comparability** |  | | | | | | | |
| Comparability of exposed and non-exposed queues | ★ | ★ | ★ | ★★ | ★ | ★ | ★ | ★ |
| **Outcome** |  | | | | | | | |
| Methods of measurement of results. | ★ | ★ | ★ | ★ | ★ | ★ | ★ | ★ |
| Follow-up time | ★ | ★ | ★ | ★ | ★ | ★ | ★ | ★ |
| Completeness of follow-up | ★ | ★ | ★ | ★ | ★ | ★ | ★ | ★ |
| **Total** | 8 | 8 | 8 | 9 | 7 | 8 | 8 | 8 |

Each five-pointed star represents 1 point, and the points are accumulated at the end. Part Comparability entries can be rated up to 2 points, and the remaining entries can be rated up to 1 point each.

Modified Jadad Scale for assessing the quality of RCT studies in meta-analysis

| **Study** | **Randomalzation(2)** | **Blinding(2)** | **Randomize hiding(2)** | **Withdrawals and dropouts(1)** | **Total** |
| --- | --- | --- | --- | --- | --- |
| Rui Zhang 2022 | 2 | 1 | 1 | 1 | 5 |
| Lili Liu 2021 | 1 | 1 | 1 | 1 | 4 |

The number in parentheses represents the highest score an entry can receive.
